# The Exported Chaperone PfHsp70x Is Dispensable for the *Plasmodium falciparum* Intraerythrocytic Life Cycle

**DOI:** 10.1128/mSphere.00363-17

**Published:** 2017-09-27

**Authors:** David W. Cobb, Anat Florentin, Manuel A. Fierro, Michelle Krakowiak, Julie M. Moore, Vasant Muralidharan

**Affiliations:** a Department of Cellular Biology, University of Georgia, Athens, Georgia, USA; b Center for Tropical and Emerging Global Diseases, University of Georgia, Athens, Georgia, USA; c Department of Infectious Diseases, University of Georgia, Athens, Georgia, USA; University at Buffalo

**Keywords:** *Plasmodium falciparum*, Hsp70, malaria, protein export

## Abstract

Export of parasite proteins into the host erythrocyte is essential for survival of *Plasmodium falciparum* during its asexual life cycle. While several studies described key factors within the parasite that are involved in protein export, the mechanisms employed to traffic exported proteins within the host cell are currently unknown. Members of the Hsp70 family of chaperones, together with their Hsp40 cochaperones, facilitate protein trafficking in other organisms, and are thus likely used by *P. falciparum* in the trafficking of its exported proteins. A large group of Hsp40 proteins is encoded by the parasite and exported to the host cell, but only one Hsp70, *P. falciparum* Hsp70x (PfHsp70x), is exported with them. PfHsp70x is absent in most *Plasmodium* species and is found only in *P. falciparum* and closely related species that infect apes. Herein, we have utilized clustered regularly interspaced short palindromic repeat (CRISPR)/Cas9 genome editing in *P. falciparum* to investigate the essentiality of PfHsp70x. We show that parasitic growth was unaffected by knockdown of PfHsp70x using both the dihydrofolate reductase (DHFR)-based destabilization domain and the *glmS* ribozyme system. Similarly, a complete gene knockout of PfHsp70x did not affect the ability of *P. falciparum* to proceed through its intraerythrocytic life cycle. The effect of PfHsp70x knockdown/knockout on the export of proteins to the host red blood cell (RBC), including the critical virulence factor *P. falciparum* erythrocyte membrane protein 1 (PfEMP1), was tested, and we found that this process was unaffected. These data show that although PfHsp70x is the sole exported Hsp70, it is not essential for the asexual development of *P. falciparum*.

**IMPORTANCE** Half of the world’s population lives at risk for malaria. The intraerythrocytic life cycle of *Plasmodium* spp. is responsible for clinical manifestations of malaria; therefore, knowledge of the parasite’s ability to survive within the erythrocyte is needed to combat the deadliest agent of malaria, *P. falciparum*. An outstanding question in the field is how *P. falciparum* undertakes the essential process of trafficking its proteins within the host cell. In most organisms, chaperones such as Hsp70 are employed in protein trafficking. Of the *Plasmodium* species causing human disease, the chaperone PfHsp70x is unique to *P. falciparum*, and it is the only parasite protein of its kind exported to the host (S. Külzer et al., Cell Microbiol 14:1784–1795, 2012). This has placed PfHsp70x as an ideal target to inhibit protein trafficking and kill the parasite. However, we show that PfHsp70x is not required for export of parasite effectors and it is not essential for parasite survival inside the RBC.

## Abstract (precis)

Half of the world’s population lives at risk for malaria. The intraerythrocytic life cycle of *Plasmodium* spp. is responsible for clinical manifestations of malaria; therefore, knowledge of the parasite’s ability to survive within the erythrocyte is needed to combat the deadliest agent of malaria, *P. falciparum*. An outstanding question in the field is how *P. falciparum* undertakes the essential process of trafficking its proteins within the host cell. In most organisms, chaperones such as Hsp70 are employed in protein trafficking. Of the *Plasmodium* species causing human disease, the chaperone PfHsp70x is unique to *P. falciparum*, and it is the only parasite protein of its kind exported to the host (S. Külzer et al., Cell Microbiol 14:1784–1795, 2012). This has placed PfHsp70x as an ideal target to inhibit protein trafficking and kill the parasite. However, we show that PfHsp70x is not required for export of parasite effectors and it is not essential for parasite survival inside the RBC.

## INTRODUCTION

Malaria is a profound killer worldwide. In 2015, 214 million cases of malaria resulted in 438,000 deaths, largely in Africa and Asia (1). Within countries where malaria is endemic, the disease targets the most-vulnerable members of the population, including children less than 5 years old and pregnant women (1). The disease is caused by infection with eukaryotic parasites from the genus *Plasmodium*, but it is one species*—Plasmodium falciparum*—that is responsible for most of the mortality associated with malaria. The clinical manifestations of malaria range from fever, headache, and muscle pains to severe anemia, coma, and respiratory distress (2). All of these symptoms are direct consequences of asexual replication of the parasite within the human red blood cell (RBC) (3). During this cycle of replication, *P. falciparum* invades the RBC and dramatically transforms its morphology and physiology. Alterations to the RBC include increased permeability, loss of cell deformability, and introduction of virulence-associated knobs at the RBC membrane (4, 5).

Remodeling of the RBC requires export of hundreds of parasite proteins into the host cell, a feat involving protein trafficking through multiple compartments before arriving at their final destinations in the host. The first phase of the journey begins in the parasite endoplasmic reticulum (ER). Many exported proteins contain an N-terminal signal motif termed the host targeting signal or *Plasmodium* export element (PEXEL) (5, 6). A key step in the export of PEXEL-containing proteins is cleavage of the motif by the ER-resident aspartyl protease plasmepsin V (7–9, 45). A subgroup of exported proteins called PEXEL-negative exported proteins (PNEPs) lack the motif, but their N termini are similarly necessary for export (10, 11). Aside from plasmepsin V processing of PEXEL, mechanisms underlying the selection of host-destined proteins for exit from the ER remain unclear. Nonetheless, PEXEL-containing proteins and PNEPs continue their journey through the parasite’s secretory pathway and are delivered to the parasitophorous vacuole (PV), a membranous structure within which the parasite resides. Previous studies have shown that proteins cross the parasitophorous vacuole membrane (PVM) through the *Plasmodium* translocon of exported proteins (PTEX) (12–14). Once they are on the other side of the PVM, all classes of proteins need to refold and find their specific subcellular localization, whether it is in the host cytoplasm, host membrane, or parasite-induced structures such as knobs or Maurer’s clefts. It is completely unknown how hundreds of proteins, within a short time period, cross through the PTEX, refold to regain structure and function, and find their final destination in the host.

The process of protein export is essential for *P. falciparum* survival in the RBC, as blockage of protein export—whether at the parasite ER or at the PVM—results in death of the parasite. In the ER, overexpression of catalytically dead plasmepsin V (PMV) results in impaired parasite growth, and inhibition of PMV with a PEXEL mimetic impairs protein export and kills parasites during the transition to the trophozoite stage (9, 15, 16). Similarly, *P. falciparum* parasites are sensitive to interference of trafficking across the PVM. Conditional knockdown of PTEX components blocks protein export and kills the parasites (17, 18). As the parasites are susceptible to inhibition of trafficking in the ER and PV, interference in the trafficking process within the host may similarly impair parasite growth. The mechanisms of protein trafficking inside the host cell remain unknown, but identification of essential components of this process will provide valuable targets for drug discovery programs.

Molecular chaperones are likely candidates in the search for key export and trafficking components. Indeed, *P. falciparum* Hsp101 (PfHsp101) is an essential component of PTEX, and its inhibition results in accumulation of exported proteins within the PV (17). Furthermore, several parasite Hsp40s are exported to the RBC, but their function there is unknown (19). In other organisms, Hsp40s serve as cochaperones for Hsp70s, but in contrast to the large number of exported Hsp40s, *P. falciparum* Hsp70x (PfHsp70x) (PF3D7_0831700) is the only parasite-encoded Hsp70 that is exported to the host cell (20, 21). This chaperone is found only in *P. falciparum* and closely related species that cause malaria in apes such as *Plasmodium reichenowi*, but not in other *Plasmodium* species that infect humans, such as *P. vivax* or *P. knowlesi* (20). Within the *P. falciparum*-infected RBC, PfHsp70x is localized to the PV and the host, where it associates with PfHsp40s in mobile structures termed J-dots (20). Given its status as the sole exported Hsp70, we hypothesized that PfHsp70x is central to protein trafficking in the host cell, and thus essential to parasite viability. Indeed, studies focused on PTEX interactions have found PfHsp70x associated with the translocon, and it has been shown to colocalize with the critical virulence protein PfEMP1 during its trafficking (20, 22, 23).

In this study, we took advantage of various genetic techniques to show that PfHsp70x is nonessential for protein export and parasite growth. We have used the dihydrofolate reductase (DHFR)-based destabilizing domain (DDD) that has previously been used to inhibit chaperone function (17, 24). In addition, we have used the *glmS* ribozyme system that inhibits translation via mRNA degradation (25). Mutants for both knockdown methods were successfully generated, but knockdown had no impact on parasite growth or protein export, including no discernible difference in the export of PfEMP1. To confirm that the lack of a phenotype was not due to incomplete knockdown, we used clustered regularly interspaced short palindromic repeat (CRISPR)/Cas9 technology to generate a complete knockout of the PfHsp70x gene and found no defects in parasite proliferation or export. Our data demonstrate that PfHsp70x is not required for protein export to the host RBC and not essential for the intraerythrocytic life cycle of *P. falciparum*.

## RESULTS

### Conditional mutants of PfHsp70x.

Previous work has shown that the DHFR-based destabilization domain (DDD) fusions can lead to the inhibition of protein-protein interactions (17, 24) or degradation of the DDD-tagged proteins (26–28). In the presence of the stabilizing ligand trimethoprim (TMP), the DDD is folded, and the chaperone functions normally. However, upon TMP removal, the DDD is unfolded and binds to its attached chaperone intramolecularly, thereby blocking interactions with the chaperone’s client proteins and inhibiting normal chaperone function (see Fig. S1A in the supplemental material). Relying on single-crossover homologous recombination, the *pfhsp70x* gene was modified with a triple-hemagglutinin (triple-HA) tag and the DDD, and integration at the *pfhsp70x* locus was confirmed via Southern blot analysis (Fig. S1A and B). Consistent with the autoinhibitory model of chaperone-DDD action, Western blot analysis of parasite lysates following TMP removal showed that PfHsp70x protein levels remain consistent over time (Fig. S1C). Isolation of the host cell cytoplasm using saponin lysis revealed that PfHsp70x-DDD is exported to the host cell (Fig. S1C). Moreover, the persistence of PfHsp70x in the supernatant following TMP removal indicated that PfHsp70x is exported to the host cell even in its putative inhibited form. To assess the role of PfHsp70x in parasite proliferation, we removed TMP and measured asexual growth over several days and at least two replication cycles. We found that the absence of TMP had no effect on parasite proliferation (Fig. S2A). It was previously reported that PfHsp70x, together with several other exported chaperones, localizes to specific punctate structures in the host cell termed J-dots. To test the effect of DDD-based inhibition on PfHsp70x localization, we performed immunofluorescence assays and found that PfHsp70x-DDD is trafficked to the expected punctate structures within the host cell, regardless of the presence of TMP (Fig. S2B). These data suggest that unlike other chaperones, PfHsp70x activity was unaffected by the DDD fusion or that inhibition of PfHsp70x using the DDD system does not affect the asexual life cycle of the parasite. We therefore utilized alternative methods to reduce PfHsp70x protein levels in the parasite.

10.1128/mSphere.00363-17.1 FIG S1  Generating PfHsp70x-DDD parasites. (A) Mechanism of PfHsp70x-DDD conditional inhibition. The *pfhsp70x* locus was modified to contain a triple-hemagglutinin (HA) tag and a DHFR-based destabilization domain (DDD). In the presence of trimethoprim (TMP), the DDD is stable, and the chaperone is active. Upon TMP removal, the chaperone binds the DDD intramolecularly and cannot interact with client proteins, inhibiting normal activity. (B, top) Single-crossover homologous recombination enables the integration of the plasmid into the 3′ end of the *pfhsp70x* gene. (Bottom) Southern blot analysis of genomic DNA isolated from parasites. The parasite lines are indicated above the lanes. The genomic DNA was digested with AccI. Bands expected from integration of the plasmid into the 3′ end of the *pfhsp70x* gene were observed in two independent transfections. A single band indicative of the parental allele was observed for the parental strain, and it was absent in the integrant parasites. (C) PfHsp70x-DDD parasites were incubated without TMP, and schizont-stage parasites were purified on a Percoll gradient. Host cell lysates together with exported proteins were isolated using 0.04% cold saponin and were then collected from the supernatant (S). Parasite cells with all nonexported proteins were collected from the pellet (P). Using Western blot analysis, the two fractions were analyzed and probed for PfHsp70x expression and export. The membrane was probed with antibodies against HA (top) and plasmepsin V (loading control) (bottom). The protein marker sizes that comigrated with the probed protein are shown on the left. Download FIG S1, TIF file, 2.4 MB. Copyright © 2017 Cobb et al. 2017 Cobb et al. This content is distributed under the terms of the Creative Commons Attribution 4.0 International license.

10.1128/mSphere.00363-17.2 FIG S2  TMP removal does not affect parasite growth and PfHsp70x localization. (A) Asynchronous PfHsp70x-DDD parasites were grown with 10 µM TMP or without TMP, and parasitemia was monitored every 24 h for 5 days. Data are fit to an exponential growth equation and are represented as means ± SEM (error bars). Experiments were done three times, and biological replicates are shown. (B) Immunofluorescence imaging of acetone-fixed PfHsp70x-DDD parasites stained with anti-HA (red) and DAPI (blue). From left to right, the images are parasites stained with anti-HA (red), parasites stained with DAPI (blue), fluorescence merge images, and phase-contrast images. Bars, 5 µm. Download FIG S2, TIF file, 1.6 MB. Copyright © 2017 Cobb et al. 2017 Cobb et al. This content is distributed under the terms of the Creative Commons Attribution 4.0 International license.

**FIG 1:**
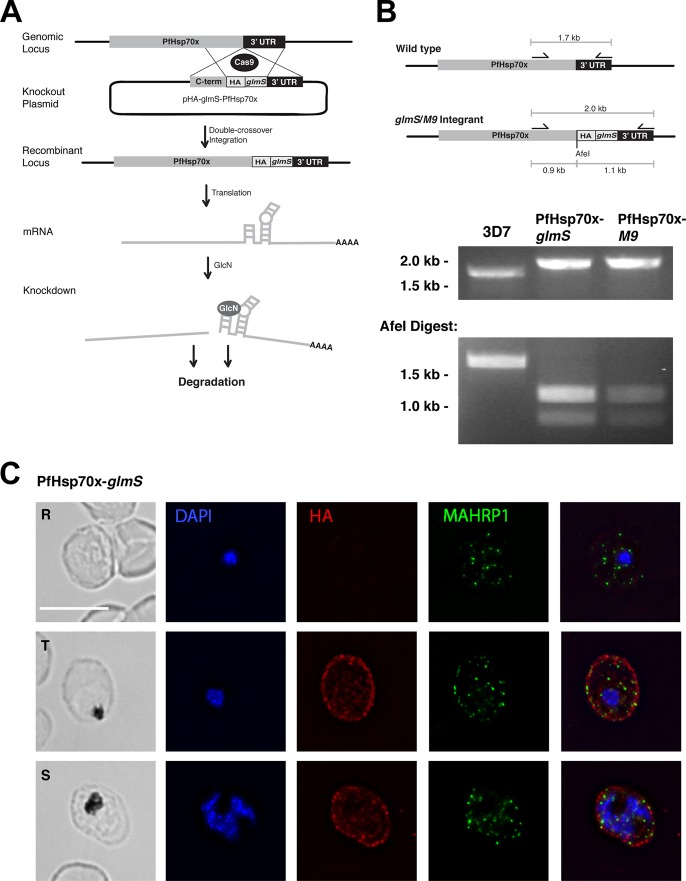
CRISPR/Cas9-mediated integration of HA-glmS/M9 at the PfHsp70x locus. (A) Diagram showing integration of the HA-ribozyme sequence and GlcN-induced degradation of mRNA. Cas9 introduces a double-stranded break at the beginning of the 3′ UTR of the *pfhsp70x* locus. The repair plasmid provides homology regions for double-crossover homologous recombination, introducing a triple-hemagglutinin (HA) tag and the ribozyme sequence. Following translation and addition of glucosamine (GlcN), the PfHsp70x-*glmS* mRNA is cleaved by the ribozyme and is subject to degradation. C-term, C terminus. (B) PCR test confirming integration at the PfHsp70x locus. DNA was purified from transfected, cloned parasites, and primers were used to amplify the region between the C terminus and the 3′ UTR of *pfhsp70x*. The PCR products were digested with AfeI, further confirming integration. (C) IFA showing export of HA-tagged PfHsp70x. Asynchronous PfHsp70x-*glmS* parasites were fixed with acetone and stained with specific antibodies. From left to right, the images are phase-contrast micrographs of parasites, parasites stained with DAPI (parasite nucleus) (blue), parasites stained with anti-HA antibody (red), parasites stained with anti-MAHRP1 antibody (green), and fluorescence merge images of the parasites. Abbreviations: R, rings; T, trophozoites; S, schizonts. Bar, 5 µm.

Next, we sought to conditionally knock down PfHsp70x at the mRNA level using the *glmS* ribozyme (25). In this system, the *glmS* ribozyme sequence is inserted into the 3′ end of the genomic locus of a gene and is transcribed with the gene as one mRNA. Addition of the small molecule glucosamine (GlcN) activates the *glmS* ribozyme, which cleaves itself from the mRNA, disconnecting the transcript from its poly(A) tail and leading to its degradation (Fig. 1A). Using CRISPR/Cas9 genome engineering, we appended a triple-HA tag to the C terminus of PfHsp70x, followed by the *glmS* ribozyme to make the PfHsp70x*-glmS* protein (Fig. 1A) (29). A second cell line was generated in which the *pfhsp70x* locus was tagged with a mutant version of the ribozyme—termed *M9*—which is unresponsive to GlcN and serves as a control during GlcN treatment (25). Following transfection and drug selection, PfHsp70x*-glmS* and PfHsp70x*-M9* clones were isolated via limiting dilutions. PCR analysis revealed the correct integration of the tag and ribozyme into the *pfhsp70x* gene in all clonal parasite lines (Fig. 1B). Additionally, immunofluorescence assays confirmed that the PfHsp70x*-glmS* protein is exported to the host cytoplasm, where it is found, as before, in punctate structures that are distinct from Maurer’s clefts, suggestive of J-dot localization (Fig. 1C).

**FIG 2:**
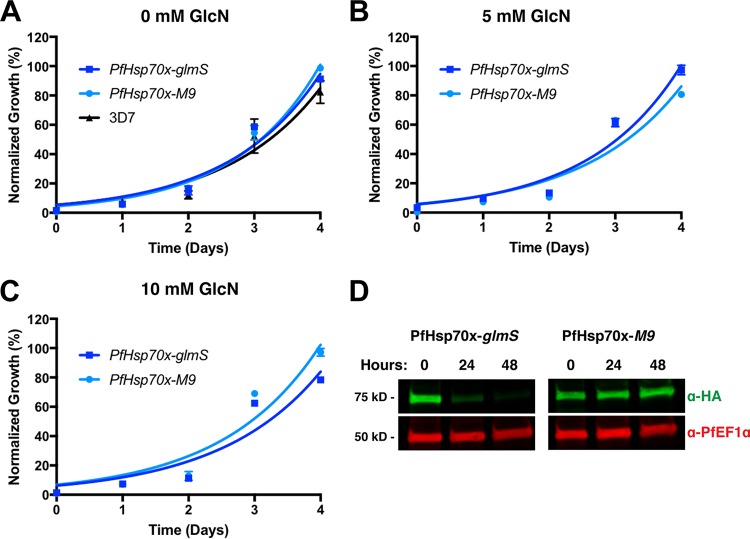
GlcN-induced knockdown of PfHsp70x does not affect intraerythrocytic growth. (A) PfHsp70x-*glmS*, PfHsp70x-*M9*, and 3D7 (parental) cell lines were seeded at equal parasitemia in triplicate and grown in normal culturing medium. Parasitemia was measured every 24 h using flow cytometry. Data are fit to an exponential growth equation and are represented as means ± standard errors of the means (SEM) (error bars) (*n* = 3). (B and C) PfHsp70x-*glmS* and PfHsp70x-*M9* parasites were seeded at equal parasitemia in triplicate. Cultures were grown in the presence of either 5 mM or 10 mM GlcN. Parasitemia was measured every 24 h using flow cytometry. Data are fit to an exponential growth equation and are represented as means ± SEM (*n* = 3). (D) PfHsp70x-*glmS* and PfHsp70x-*M9* parasites were grown in the presence of 7.5 mM GlcN. Schizont-stage parasites were purified on a Percoll gradient every 24 h, and whole-parasite lysates were used for Western blot analysis. The membrane was probed with anti-HA (α-HA) and anti-PfEF1α (loading control) antibodies. The positions of molecular mass markers (in kilodaltons) are indicated to the left of the blot.

Next, we tested the effects of reducing PfHsp70x levels on intraerythrocytic growth. To ensure that insertion of the ribozyme itself does not interfere with normal asexual growth, the PfHsp70x*-glmS* and PfHsp70x*-M9* cell lines and the parental line (3D7) were grown in the absence of GlcN. Indeed, we found that in the absence of GlcN, growth of both the *glmS* and *M9* cell lines was comparable to the growth of 3D7 (Fig. 2A). Next, the PfHsp70x*-glmS* and PfHsp70x-*M9* cell lines were cultured with GlcN, and parasitemia was measured via flow cytometry. The growth of PfHsp70x*-glmS* and PfHsp70x*-M9* cell lines was unaffected by treatment with 5 mM and 10 mM GlcN (Fig. 2B and C). To confirm that the level of PfHsp70x protein is reduced in response to GlcN, schizont-stage parasites from the *glmS* and *M9* cell lines were purified with Percoll, and whole-parasite lysates were used for Western blotting. Using anti-HA antibody, we found that treatment with GlcN reduced protein levels in the PfHsp70x*-glmS* cell line but did not affect protein levels in the PfHsp70x*-M9* cell line (Fig. 2D). Together, these data show that we can efficiently reduce PfHsp70x levels using the *glmS* ribozyme, but this has no effect on the asexual growth of the parasite within the RBC.

### Protein export is unimpaired in PfHsp70x knockdown parasites.

**FIG 3:**
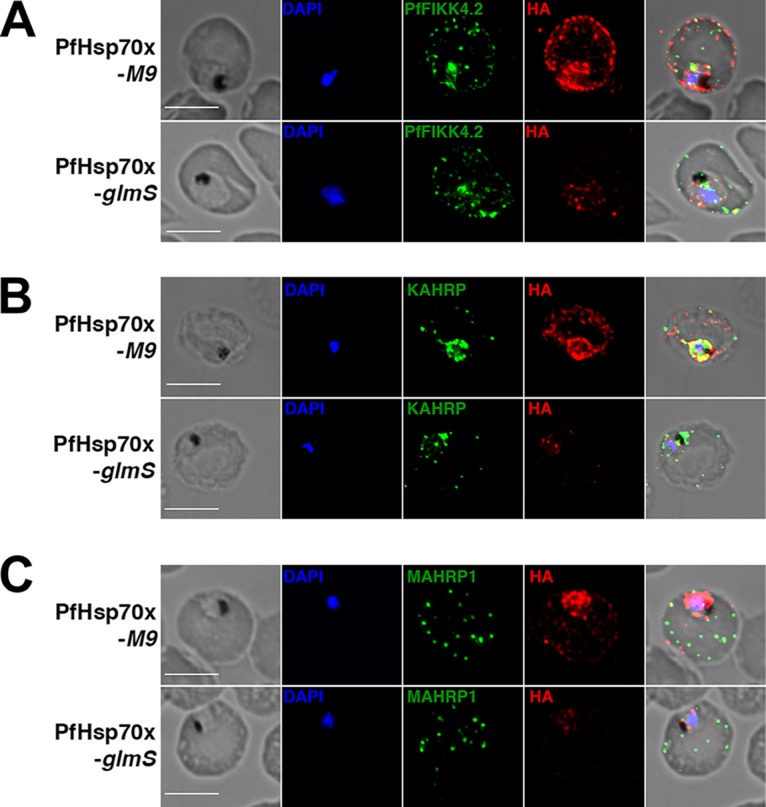
PfHsp70x knockdown does not inhibit export of virulence-associated proteins. Asynchronous PfHsp70x-*M9* and PfHsp70x-*glmS* parasites were fixed with acetone (PfFIKK4.2 and MAHRP1) or paraformaldehyde (KAHRP) and stained with antibodies against PfFIKK4.2 (A), KAHRP (B), or MAHRP1 (C). DAPI was used to mark parasite cell nucleus. From left to right, the images are phase-contrast micrographs of parasites, parasites stained with DAPI (blue), parasites stained with antibody against the exported protein (green), parasites stained with anti-HA antibody (red), and fluorescence and phase-contrast merge images of the parasites. Representative images are shown. Bars, 5 µm.

Although parasite growth was unaffected by PfHsp70x knockdown, we reasoned that it could nonetheless play a role in export of proteins to the host cell. In particular, we hypothesized that PfHsp70x is needed for the export of proteins known to mediate virulence of *P. falciparum* infection, as trafficking defects of these proteins would not manifest as arrest of the asexual life cycle (19). Using immunofluorescence, we examined localization of specific virulence-associated proteins in PfHsp70x-*M9* and PfHsp70x-*glmS* parasites after 72 h of growth in GlcN-supplemented medium. First, the localization of the PEXEL-containing PfFIKK4.2, an exported kinase associated with knob formation and infected RBC rigidity, is unchanged in control versus PfHsp70x knockdown parasites (Fig. 3A) (30). Next, we examined the localization of the PEXEL-containing protein KAHRP (knob-associated histidine-rich protein), which is essential for the formation of knobs on the surfaces of infected RBCs (31). Export of this protein was not inhibited in PfHsp70x knockdown parasites (Fig. 3B). Finally, we determined the localization of the PNEP MAHRP1 (Maurer’s cleft histidine-rich protein 1), which has been implicated in the presentation of antigenically variant proteins, including PfEMP1, at the RBC surface, and we found that its export is not impaired by the knockdown of PfHsp70x (Fig. 3C) (32). As demonstrated by HA staining of Western blots and indirect immunofluorescence assay (IFA) (Fig. 2D and Fig. 3), PfHsp70x is reduced, but not completely ablated, using the *glmS* ribozyme. We reasoned that the reduced level of PfHsp70x that is produced during GlcN treatment could be sufficient for parasite survival, and therefore endeavored next to knock out *pfhsp70x*.

### Knockout of *pfhsp70x* does not affect parasite growth.

**FIG 4:**
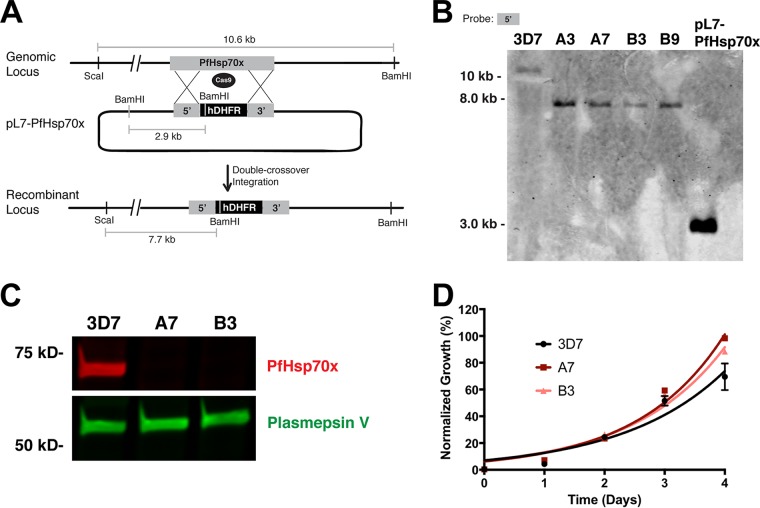
Knockout of *pfhsp70x* does not affect intraerythrocytic growth. (A) Schematic representation showing interruption of the PfHsp70x ORF with the *hDHFR* cassette. The Cas9-mediated double-stranded break in the *pfhsp70x* ORF is repaired using homology regions on the template plasmid while inserting an *hDHFR* cassette into the locus. (B) Southern blot analysis confirming knockout of PfHsp70x. Genomic DNA from independent knockout clones (A3, A7, B3, and B9) was isolated and digested with BamHI and ScaI. The membrane was hybridized with a biotin-labeled probe complementary to the first 800 bp of the *pfhsp70x* ORF. (C) Western blot analysis demonstrating loss of PfHsp70x protein expression in independent knockout clones. Schizont-stage parasites were purified on a Percoll gradient, and whole-cell lysate was used for analysis. The membrane was probed with antibody raised against PfHsp70x and antibody against plasmepsin V as a loading control. (D) Parental lines and independent PfHsp70x-KO clones (A7 and B3) were seeded at equal parasitemia in triplicate. Parasitemia was measured every 24 h using flow cytometry. Data are fit to an exponential growth equation and are represented as means ± SEM (*n* = 3).

We utilized two different conditional knockdown systems to modify the PfHsp70x locus, but these approaches were insufficient to produce a growth defect in the parasites. Therefore, we sought to definitively test the essentiality of PfHsp70x via complete genomic knockout (KO). To this end, we employed CRISPR/Cas9 to interrupt the PfHsp70x open reading frame (ORF) by inserting a human dihydrofolate reductase (*hdhfr*) drug resistance cassette (Fig. 4A). Following transfection and selection with WR99210, PfHsp70x-KO parasites were cloned via limiting dilutions. Southern blot analysis of genomic DNA isolated from the parental line and independent clones showed that the *hdhfr* cassette was inserted into the *pfhsp70x* gene via homology-directed repair (Fig. 4B). To verify that the null mutants do not express PfHsp70x, schizont-stage parasites from two independent knockout clones and the parental line were purified on a Percoll gradient, and whole-parasite lysates were used for Western blotting. Probing with anti-PfHsp70x shows that the knockout clones do not express PfHsp70x (Fig. 4C). Intraerythrocytic growth of the PfHsp70x-KO clones was monitored over two replication cycles. In agreement with the lack of any growth phenotype in the conditional knockdown parasite lines, the PfHsp70x-KO parasites displayed wild-type level of proliferation in erythrocytes (Fig. 4D). Finally, we measured the susceptibility of PfHsp70x-KO clones to heat shock stress by monitoring their growth after a heat shock (Fig. S3). These data show that the PfHsp70x-KO parasites are able to deal with heat shock just as well as the wild-type parasites (Fig. S3). The normal growth in the complete absence of PfHsp70x expression conclusively demonstrates that PfHsp70x activity is not essential for the asexual growth of the parasite within the RBC.

10.1128/mSphere.00363-17.3 FIG S3  Heat shock does not inhibit the growth of PfHsp70x-KO parasites. 3D7 and PfHsp70x-KO clones A7 and B3 were subjected to 40°C heat shock for 4 h, and parasitemia was measured every 24 h using flow cytometry. Data are fit to an exponential growth equation and are represented as means ± SEM (*n* = 3). Download FIG S3, TIF file, 0.2 MB. Copyright © 2017 Cobb et al. 2017 Cobb et al. This content is distributed under the terms of the Creative Commons Attribution 4.0 International license.

### Protein export is unimpaired in PfHsp70x-KO parasites.

**FIG 5:**
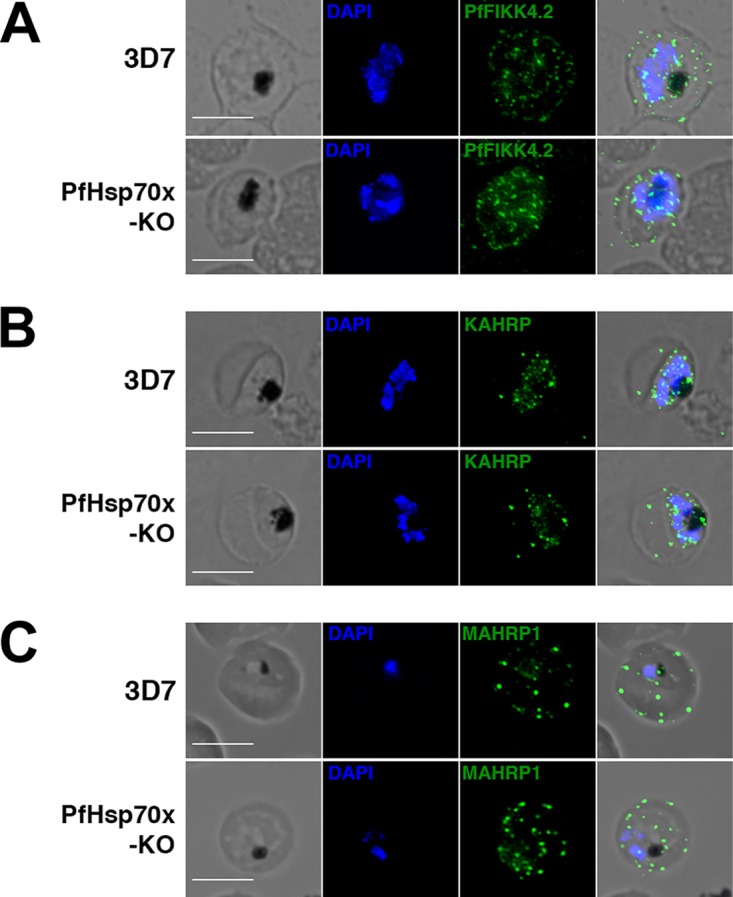
PfHsp70x knockout does not inhibit export of virulence-associated proteins. Asynchronous 3D7 and PfHsp70x-KO parasites were fixed with acetone (PfFIKK4.2 and MAHRP1) or paraformaldehyde (KAHRP) and stained with antibodies against PfFIKK4.2 (A), KAHRP (B), or MAHRP1 (C). DAPI was used to mark parasite cell nucleus. From left to right, the images are phase-contrast micrographs of the parasites, parasites stained with DAPI (blue), parasites stained with antibody against exported protein (green), and fluorescence and phase-contrast merge. Representative images are shown. Bars, 5 µm.

Using PfHsp70x-KO parasites, we next tested the hypothesis that the chaperone is required for export of virulence-associated proteins. Using immunofluorescence, we examined the export of the same proteins assayed with PfHsp70x-*glmS* parasites: PfFIKK4.2, KAHRP, and MAHRP1 (30–32). Consistent with our observations using PfHsp70x-*glmS*, *pfhsp70x* knockout did not interrupt export of these proteins (Fig. 5). These data show that the loss of PfHsp70x does not impede the parasite’s ability to export virulence-associated proteins to the host cell.

### Export of antigenic proteins to the host RBC is unaffected in PfHsp70x mutants.

**FIG 6:**
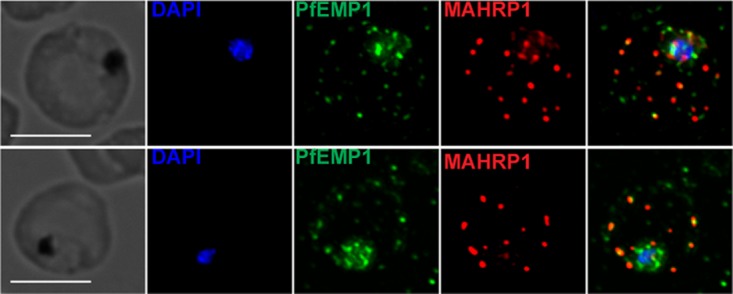
PfHsp70x knockout does not inhibit export of PfEMP1 to the host cell. Asynchronous 3D7 and PfHsp70x-KO parasites were fixed with acetone and stained with antibodies against the ATS domain of PfEMP1 and MAHRP1. DAPI was used to stain parasite cell nucleus. The images from left to right are phase, DAPI (blue), PfEMP1 (green), MAHRP1 (red), and fluorescence merge. Representative images are shown.

**FIG 7:**
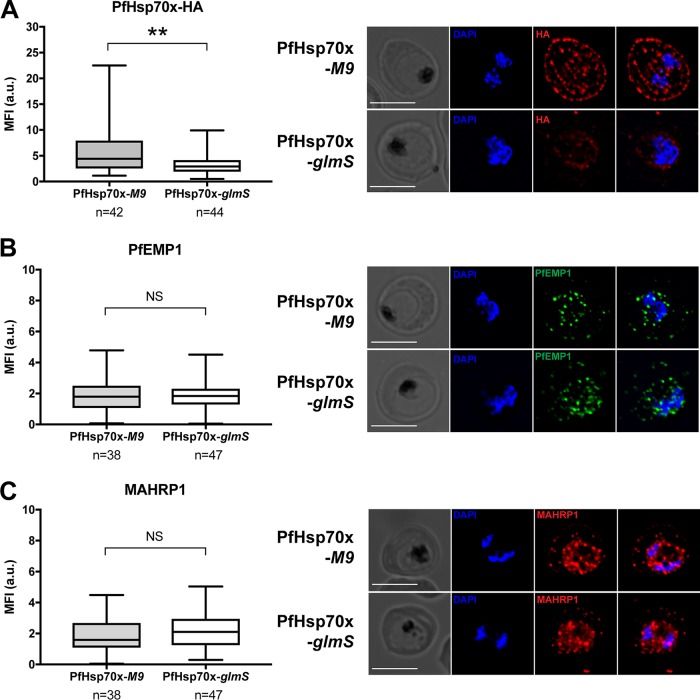
Knockdown of PfHsp70x does not inhibit export of PfEMP1 to the host cell. (A to C) PfHsp70x-*M9* and PfHsp70x-*glmS* parasites were fixed with acetone and stained with antibodies against HA, PfEMP1, or MAHRP1. DAPI was used to mark parasite cell nucleus. (Right) From left to right, the images are phase-contrast micrographs of parasites, parasites stained with DAPI, parasites stained with anti-HA antibody or antibody against exported protein, and fluorescence merge image. Representative images are shown. Bars, 5 µm. (Left) The mean fluorescence intensity (MFI) for each protein was calculated for individual cells and shown as box-and-whisker plots, with whiskers representing the maximum and minimum MFI. For HA, the MFI was calculated for the entire infected RBC. For PfEMP1 and MAHRP1, MFI was calculated for the exported fraction only. Significance was determined using an unpaired *t* test (**, *P* ≤ 0.01; NS, not significant).

PfHsp70x was shown to interact with the antigenically variant protein PfEMP1, and recent data that identified proteins that interact with PfEMP1 confirm these results (20). Therefore, we wanted to test how the export of PfEMP1 is affected in our mutants. Utilizing immunofluorescence microscopy, we determined the localization of PfEMP1 in 3D7 and PfHsp70x-KO parasites (Fig. 6). Our data show that knockout of PfHsp70x does not prevent export of PfEMP1 to the host cell (Fig. 6). Next, we observed the export of PfEMP1 in our PfHsp70x conditional mutants. Our data show that PfEMP1 is exported equally well in both PfHsp70x-*M9* and PfHsp70x-*glmS* parasites under knockdown conditions (Fig. 7). We quantified the amount of PfHsp70x-HA, as well as the amount of exported PfEMP1, in these mutants and found no difference in regard to PfEMP1, despite achieving significant reduction of PfHsp70x in the *glmS* parasite line (Fig. 7A and B). Because MAHRP1 has been implicated in the trafficking of PfEMP1, we also quantified the export of MAHRP1 in the PfHsp70x conditional mutants, and we found that knockdown of PfHsp70x does not affect MAHRP1 export (Fig. 7C).

**FIG 8:**
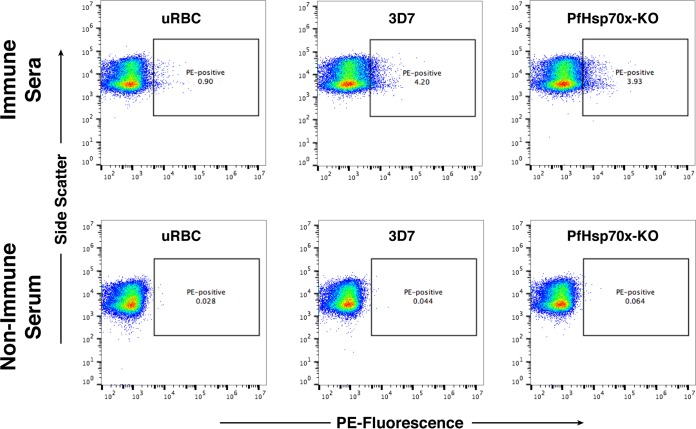
Human immune sera recognizes 3D7 and PfHsp70x-KO parasites. Synchronized 3D7 and PfHsp70x-KO parasites were incubated with either pooled human sera from Kenya where malaria is endemic (top panels) or pooled nonimmune human serum from the United States (bottom panels). Recognition by the serum was determined using a PE-conjugated anti-human IgG antibody and flow cytometry. Uninfected red blood cells (uRBC) were also assayed. Side scatter is shown on the *y* axes, and PE fluorescence is shown on the *x* axes.

Next, we sought to investigate whether there were any differences in the mutants in the export of antigenic parasite proteins that generate an immune response. We obtained pooled human sera collected from a region where malaria is endemic (Kenya) as well as a region where it is not endemic (United States) (33). Uninfected RBCs, 3D7 parasites, and PfHsp70x-KO parasites were labeled with these sera and observed via flow cytometry (Fig. 8). 3D7 and PfHsp70x-KO schizonts were synchronized and grown to the schizont stage, and cultures were brought to identical parasitemia prior to labeling with sera. Our data show that both 3D7 and PfHsp70x-KO parasites are labeled equally well by human sera collected from regions where malaria is endemic but not by sera obtained from regions where malaria is not endemic, suggesting that the export of antigenic parasite proteins to the host RBC is unaffected by the loss of PfHsp70x (Fig. 8).

## DISCUSSION

While this work was under review, another study was published showing that knockout of PfHsp70x did not affect parasite growth (34). In agreement with these data, our data also demonstrate that PfHsp70x is not required for intraerythrocytic growth, even though PfHsp70x is the only parasite-encoded Hsp70 that is exported to the RBC (Fig. 2A, B, and C and Fig. 4D; also see Fig. S2A and Fig. S3D in the supplemental material). Using two different genetic approaches, we demonstrate that the export of several parasite effectors are unaffected by the loss of PfHsp70x (Fig. 3 and Fig. 5 to 8). In the case of PfEMP1, the newly published work suggests that knockout of PfHsp70x led to delays in its export and minor loss in cytoadherence, suggesting a role for PfHsp70x in parasite virulence (34). In this case, the data show that PfHsp70x knockout parasites overexpress some exported proteins (34). This suggests that there may be compensatory mechanisms that are activated when PfHsp70x is knocked out and therefore lead to minor, if any, changes in the export of parasite virulence factors (34). However, this interpretation is clouded by the lack of a conditional mutant for PfHsp70x, which cannot compensate for the loss of PfHsp70x. The data described in this study show that in both PfHsp70-KO and PfHsp70x-*glmS* mutants, export of parasite virulence factors is not affected (Fig. 3 and Fig. 5 to 8). We specifically tested the export of the antigenically variant protein, PfEMP1, which is responsible for cytoadherence, and observed that the export of PfEMP1 was unaffected in either the knockout or conditional mutants of PfHsp70x (Fig. 6 to 8). Therefore, our data suggest a slightly different, though not mutually exclusive, model than the one proposed by Charnaud et al. (34). PfHsp70x is not the only Hsp70 found in infected RBCs. Several human chaperones, including Hsp70, are present in the erythrocyte cytoplasm (35). Thus, the role played by PfHsp70x in the parasite’s biology could be redundant with the human Hsp70 that is already present in the host cell. In fact, infection with *P. falciparum* affects the normal localization of the human Hsp70, as the protein is soluble in nonparasitized RBCs but is found in detergent-resistant fractions following infection (36). Another paper published while this work was under review identified several interacting partners of PfEMP1 using thorough proteomic and genetic data (37). They identified several human chaperones, specifically from the TRiC chaperonin complex, that interact with PfEMP1. Together with our data, this suggests a model wherein PfEMP1 export is aided both by PfHsp70x and by human chaperones present in the host RBCs. This further suggests that loss of either one of them may not be enough to derail the export of parasite virulence proteins to the host RBC. The methods used here to investigate the function of PfHsp70x, knockdown and complete genomic knockout, are more challenging to use for human chaperones such as Hsp70 or the TRiC chaperonin complex. The mature RBC cannot be genetically manipulated, and knockdown of human Hsp70 in hematopoietic stem cells abrogates RBC formation (38). Our data demonstrate that pooled human sera collected from regions where malaria is endemic are unable to differentiate between wild-type and PfHsp70x-KO parasites, raising the possibility that PfHsp70x may not be required in human infections (Fig. 8). However, further detailed analysis of the *pfhsp70x* locus in strains isolated from the field or testing its role in other stages of the parasite life cycle may be informative about the essentiality of PfHsp70x in human infections. Overall, our data demonstrate that PfHsp70x is not required for export of *P. falciparum* effector proteins to the host and is dispensable for asexual growth within human RBCs and suggest a model where both human chaperones and parasite chaperones act in a redundant manner to ensure export of parasite virulence factors to the host RBCs.

## MATERIALS AND METHODS

### Plasmid construction.

Genomic DNA was isolated from *P. falciparum* using the QIAamp DNA blood kit (Qiagen). Constructs utilized in this study were confirmed by sequencing. PCR products were inserted into the respective plasmids using the In-Fusion cloning system (Clontech) or using the sequence- and ligation-independent cloning (SLIC) method. Briefly, insert and cut vector were mixed with a T4 DNA polymerase and incubated for 2.5 min at room temperature, followed by 10-min incubation on ice, and then transformed into bacteria. For generation of plasmid PfHsp70x-HADB, a 1-kb homologous sequence from the 3′ end of the *pfhsp70x* gene (not including the stop codon) was amplified by PCR using primers 5′ CACTATAGAACTCGAGGTGAAAAAGCTAAACGTGTATTATCATCATCCGCACAAGC 3′ and 5′ CGTATGGGTACCTAGGATTTACTTCTTCAACGGTTGGTCCATTATTTTGTGC 3′ and inserted into pHADB (16) using restriction sites XhoI and AvrII (New England Biolabs).

For the generation of the *glmS* conditional mutants, three plasmids were used. (i) pUF1-Cas9 (from J. J. Lopez-Rubio) was used to drive cas9 expression (29). (ii) pMK-U6 was used to drive expression of the RNA guide. For this purpose, pL6 plasmid (from J. J. Lopez-Rubio [29]) was digested with NotI and NcoI (New England Biolabs), and the fragment that contained the U6 RNA expression cassette was blunted and religated to form the pMK-U6 plasmid. The guide RNA, oligonucleotides 5′ TAAGTATATAATATTTGCATTATTGTTGTATATTTGTTTTAGAGCTAGAA 3′ and 5′ TTCTAGCTCTAAAACAAATATACAACAATAATGCAAATATTATATACTTA 3′ were annealed and cloned into the RNA module in MK-U6 as previously described (29). Briefly, pMK-U6 was digested with BtgZI (New England Biolabs), and annealed oligonucleotides were inserted using In-Fusion HD Cloning kit (Clontech). (iii) pHA-glmS and pHA-M9 were used as donor DNA templates consisting of two homology regions flanking the hemagglutinin (HA) tag and the *glmS* (or the *M9*) sequences. To generate the pHA-glmS and pHA-M9 plasmids, primers 5′ GAGCTCGCTAGCAAGCTTGCCGGCAAGATCATGTGATTTCTCTTTGTTCAAGGAGTC 3′ and 5′ TCCGCGGAGCGCTACTAGTTACCCATACGATGTTCCAGATTACGCTTACCCATACGATGTTCCAGATTACGCTTACCCATACGATGTTCCAGATTACGCTTAAATGTCCAGACCTGCAGTAATTATCCCGCCCGAACTAAGCGC 3′ were used to amplify the *glmS* and *M9* sequences from pGFP-glmS and pGFP-M9, respectively (from P. Shaw [25]). PCR constructs were then inserted into a TOPO cloning vector (Thermo Fisher). To allow efficient genomic integration of the pHA-glmS and pHA-M9 donor plasmids, 800-bp sequences were used for each homology region. The C terminus of the *pfhsp70x* coding region was PCR amplified from genomic DNA using primers 5′ AATTCGCCCTTCCGCGGGCTGTACAAGCAGCCATCTTATCAGGTGATCAATCATC 3′ and 5′ ATCGTATGGGTAAGCGCTATTTACTTCTTCAACGGTTGGTCCATTATTTTGTGCTTC 3′ and inserted into pHA-glmS and pHA-M9 using restriction sites SacII and AfeI (New England Biolabs). The 3′ untranslated region (3′ UTR) of *pfhsp70x* was PCR amplified from genomic DNA using primers 5′ ATGATCTTGCCGGCAAGCTTACGAAAATATACAACAATAATGCATAAAATAATAATAATT 3′ and 5′ CCTTGAGCTCGCTAGCGCAATATAAATGGATTATTCCTTTTGTATATAATTTAAAATAAG 3′ and inserted into pHA-glmS and pHA-M9 (already containing the C-terminal homology region) using restriction sites HindIII and NheI (New England Biolabs).

For the generation of *pfhsp70x-ko* parasites, two plasmids were used: (i) a cas9-expressing plasmid (as described above), and (ii) pL7-PfHsp70x plasmid that is derived from the pL6 plasmid (from J. J. Lopez-Rubio [29]). pL7-PfHsp70x contained the guide RNA and 800-bp homology regions flanking an *hdhfr* gene that confers resistance to WR99210. The N terminus of the *pfhsp70x* gene was amplified via PCR from genomic DNA using primers 5′ cggggaggactagtATGAAGACAAAAATTTGTAGTTATATTCATTATATTG 3′ and 5′ acaaaatgcttaagGGAAACATCTTTACCTCCATTTTTTTTTTTAAAATCTTGTAC 3′ (lowercase nucleotides are not part of the *pfhsp70x* gene but are part of the plasmid used for sequence and ligation independent cloning [SLIC]) and inserted into pL6 using restriction sites AflII and SpeI (New England Biolabs). The C terminus of the *pfhsp70x* gene was PCR amplified from genomic DNA using primers 5′ taaatctagaattcTGATCAATCATCAGCTGTCAAAGACTTATTATTATTAGATG 3′ and 5′ ttaccgttccatggTTAATTTACTTCTTCAACGGTTGGTCCATTATTTTGTGCTTC 3′ and inserted into pL6 (already containing the C-terminal homology region) using restriction sites NcoI and EcoRI (New England Biolabs). In order to insert the guide DNA sequence, oligonucleotides 5′ TAAGTATATAATATTGTACAAGCAGCCATCTTATCGTTTTAGAGCTAGAA 3′ and 5′ TTCTAGCTCTAAAACGATAAGATGGCTGCTTGTACAATATTATATACTTA 3′ were annealed and cloned into pL6 as previously described (29). Briefly, pL6 was digested with BtgZI (New England Biolabs), and annealed oligonucleotides were inserted using In-Fusion HD cloning kit (Clontech).

### Cell culture and transfections.

Parasites were cultured in RPMI 1640 medium supplemented with Albumax I (Gibco) and transfected as described earlier (39, 40). For generation of *PfHsp70x-DDD* parasites, PfHsp70x-HADB was transfected in duplicate into 3D7-derived parental strain PM1KO (KO stands for knockout) which contains a human dihydrofolate reductase (*hDHFR*) expression cassette conferring resistance to trimethoprim (TMP) (41). Selection and drug cycling were performed as described previously (24) in the presence of 10 μM TMP (Sigma). Integration was detected after three rounds of drug cycling with blasticidin (Sigma).

For generation of PfHsp70x*-glmS* and PfHsp70x*-M9* parasites, a mix of three plasmids (40 µg of each) was transfected in duplicate into 3D7 parasites. The plasmid mix contained pUF1-Cas9 (from J. J. Lopez-Rubio [29]) which contains the *DHOD* resistance gene, pMK-U6-PfHsp70x, pHA-glmS-PfHsp70x, or pHA-M9-PfHsp70x, which are all marker-free. Drug pressure was applied 48 h posttransfection, using 1 μM (DSM1) (42), selecting only for Cas9 expression. Drug was removed from the culturing medium once the parasites were detected in the culture, usually around 3 weeks posttransfection.

For generation of PfHsp70x-KO parasites, a mix of pUF1-Cas9 (from J. J. Lopez-Rubio [29]) and pL7-PfHsp70x (50 µg of each plasmid) was transfected in duplicate into 3D7 parasites. Drug pressure was applied 48 h posttransfection, using 2.5 nM WR99210 (Sigma), selecting for integration of the drug resistance cassette into the *pfhsp70x* gene.

### Growth assays.

For asynchronous growth assays of PfHsp70x-DDD lines, parasites were washed twice and incubated without TMP. For asynchronous growth assays of PfHsp70x*-glmS* and PfHsp70x*-M9* parasites, 5 or 10 mM glucosamine (GlcN) (Sigma) was added to the growth medium. Asynchronous growth assays of PfHsp70x-KO parasites were performed in medium containing WR99210. Parasitemia was monitored every 24 h via flow cytometry. For flow cytometry, aliquots of parasite cultures (5 μl) were stained with 1.5 mg/ml acridine orange (Molecular Probes) in phosphate-buffered saline (PBS). The fluorescence profiles of infected erythrocytes were measured by flow cytometry on a CyAn ADP (Beckman Coulter) or CytoFLEX (Beckman Coulter) instrument and analyzed by FlowJo software (Treestar, Inc.). Whenever required, parasites were subcultured to avoid high parasite density, and relative parasitemia at each time point was back-calculated based on actual parasitemia multiplied by the relevant dilution factors. One hundred percent parasitemia was determined as the highest relative parasitemia and was used to normalize parasite growth. Data were fit to exponential growth equations using Prism (GraphPad Software, Inc.).

### Southern blotting.

Southern blotting was performed with genomic DNA isolated using the Qiagen Blood and Cell Culture kit. Ten micrograms of DNA was digested overnight with NcoI/XmnI for PfHsp70x-DDD and BamHI/ScaI for PfHsp70x-KO (New England Biolabs). Integrants were screened using biotin-labeled probes against the 3′ end (PfHsp70x-DDD parasites) or 5′ end (PfHsp70x-KO parasites) of the *pfhsp70x* open reading frame (ORF). Southern blotting was performed as described earlier (43). The probe was labeled using biotinylated biotin-16-dUTP (Sigma). The biotinylated probe was detected on blots using IRDye 800CW streptavidin-conjugated dye (LICOR Biosciences) and imaged, processed, and analyzed using the Odyssey infrared imaging system software (LICOR Biosciences).

### Western blotting.

Western blotting was performed as described previously (26). Briefly, late-stage parasites were isolated on a Percoll gradient (Genesee Scientific). For PfHsp70x-DDD parasites, host red blood cells (RBCs) were permeabilized selectively by treatment with ice-cold 0.04% saponin in PBS for 10 min. Supernatants were collected for detection of exported parasite proteins, and pellets were collected for detection of proteins with the parasite. For PfHsp70x-KO, PfHsp70x*-glmS*, and PfHsp70x*-M9* parasites, whole-parasite lysates, including the host RBCs, were used to detect protein expression and export. The antibodies used in this study were rat anti-HA (3F10; Roche) (diluted 1:3,000), rabbit anti-PfEF1α (from D. Goldberg) (1:2,000), mouse anti-plasmepsin V (from D. Goldberg, 1:400), and rabbit anti-PfHsp70x (from J. Przyborski) (1:1,000). The secondary antibodies that were used are IRDye 680CW goat anti-rabbit IgG and IRDye 800CW goat anti-mouse IgG (LICOR Biosciences) (1:20,000). The Western blot images were processed and analyzed using the Odyssey infrared imaging system software (LICOR Biosciences).

### Microscopy and image processing.

For detection of HA tags, PfHsp70x, PfFIKK4.2, and MAHRP1, cells were smeared on a slide and fixed with acetone. For KAHRP detection, cells were fixed with paraformaldehyde and glutaraldehyde. PfHsp70x-HA was detected using rat anti-HA antibody (clone 3F10; Roche) (1:100). MAHRP1 was detected using rabbit anti-MAHRP1 (from Hans-Peter Beck) (1:500). PfFIKK4.2 and KAHRP were detected using mouse anti-PfFIKK4.2 (1:1,000) and mouse anti-KAHRP (1:1000 and 1:500, respectively; both antibodies acquired from David Cavanagh and EMRR). PfEMP1 was detected using mouse anti-ATS (1B/98-6H1-1; 1:100; Alan Cowman). Secondary antibodies used were anti-rat antibody conjugated to Alexa Fluor 488 or 594, anti-rabbit antibody conjugated to Alexa Fluor 488, and anti-mouse antibody conjugated to Alexa Fluor 488 (Life Technologies) (1:100). Cells were mounted on ProLong diamond with 4′,6′-diamidino-2-phenylindole (DAPI) (Invitrogen) and imaged using a DeltaVision II microscope system with an Olympus IX-71 inverted microscope using a 100× objective. Image processing, analysis, and display were performed using SoftWorx and Adobe Photoshop. Adjustments to brightness and contrast were made for display purposes. For quantification of PfHsp70x-HA fluorescence, PfEMP1 export, and MAHRP1 export, PfHsp70x-*glmS* and PfHsp70x-*M9* parasites were grown in the presence of 7.5 mM GlcN for 72 h, then fixed and stained with anti-HA, anti-ATS, and anti-MAHRP1 as described above. Cells were imaged as described above. The mean fluorescence intensity (MFI) for each protein was calculated as described (9). Briefly, ImageJ was used to calculate the MFI for the whole infected RBC (PfHsp70x) or the infected RBC minus the parasite in order to quantify the exported fraction (PfEMP1and MAHRP1). Differential interference contrast (DIC) images were used to exclude the parasite from analysis when calculating the MFI of the PfEMP1 and MAHRP1 exported fraction. Data were plotted using Prism (GraphPad Software, Inc.).

### Human serum staining.

3D7 and PfHsp70x-KO parasites were synchronized to the ring stage by incubating infected RBCs with 5% d-sorbitol (Amresco, Inc.) for 10 min at 37°C. The parasites were washed three times with culture medium and then allowed to proceed through the life cycle to the schizont stage. The cultures were incubated 1:10 with either pooled immune sera from Kenya or nonimmune serum from the United States for 30 min at 37°C with shaking on an orbital shaker at 880 rpm. All study procedures and instruments involving human subjects, data and sample collection, processing, and testing were approved by the University of Georgia and Centers for Disease Control and Prevention Institutional Review Boards and the Kenya Medical Research Institute Ethical Review Board. All participants provided informed, written consent under the auspices of these approved protocols (33). The serum was washed from the parasites three times with culture medium, and goat-anti-human IgG Fc conjugated to phycoerythrin (PE) was added to the parasites (1:500) (Fisher Scientific, 50-112-8944). The secondary antibody was incubated with the parasites for 30 min at 37°C with shaking. The parasites were washed three times with culture medium and resuspended in PBS, fluorescence was measured with a flow cytometer (CytoFLEX; Beckman Coulter), and data were analyzed using FlowJo software (Treestar, Inc.). Immune serum samples were collected as described above, and all samples have been deidentified (33, 44).
