# Supplementary figures and images for: The Exported Chaperone PfHsp70x Is Dispensable for the Plasmodium falciparum Intraerythrocytic Life Cycle
Source: mSphere. 2017 Sep 27;2(5):e00363-17. doi: 10.1128/mSphere.00363-17 (PMC5615134; doi:10.1128/mSphere.00363-17)

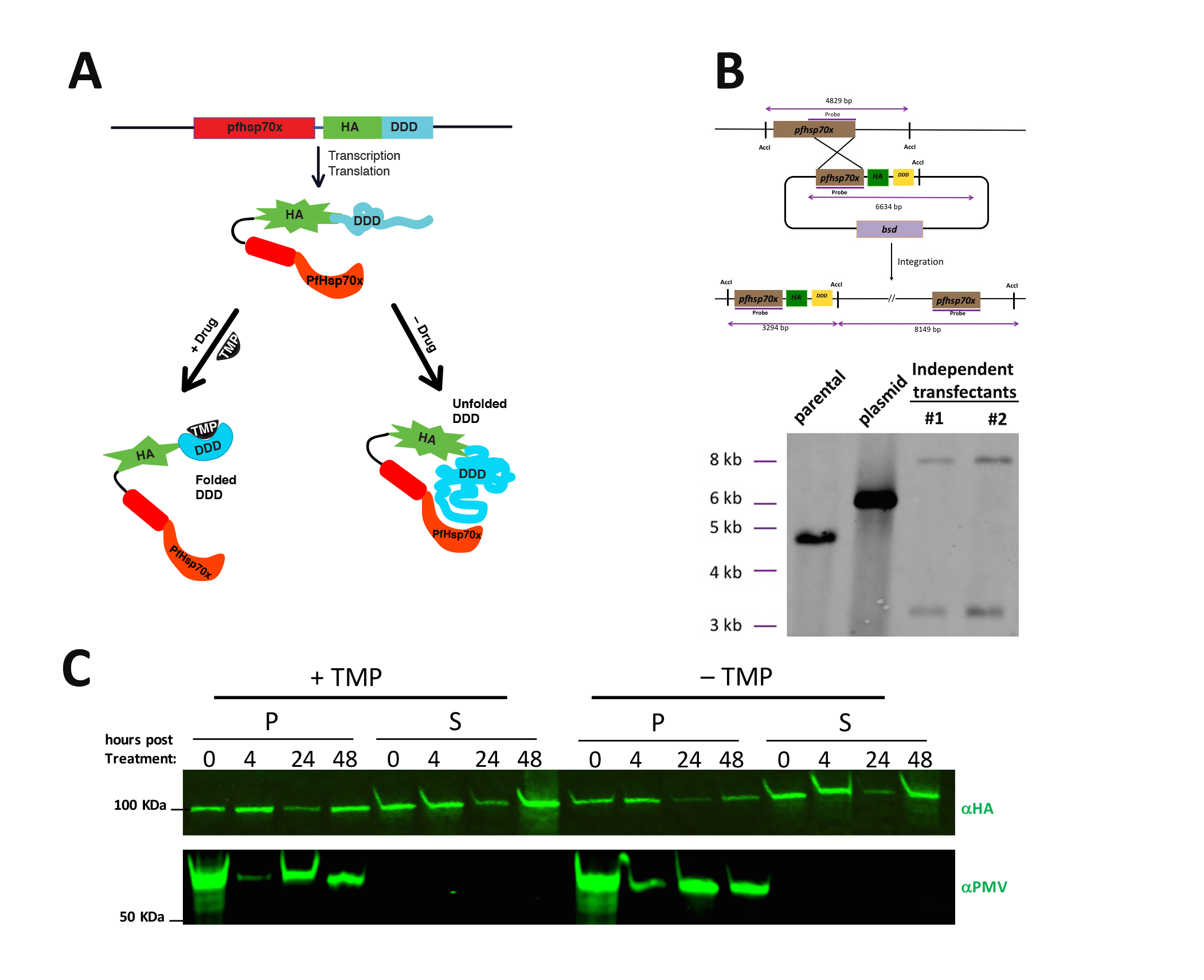

Supplement: FIG S1 [file sph005172367sf1.tif]

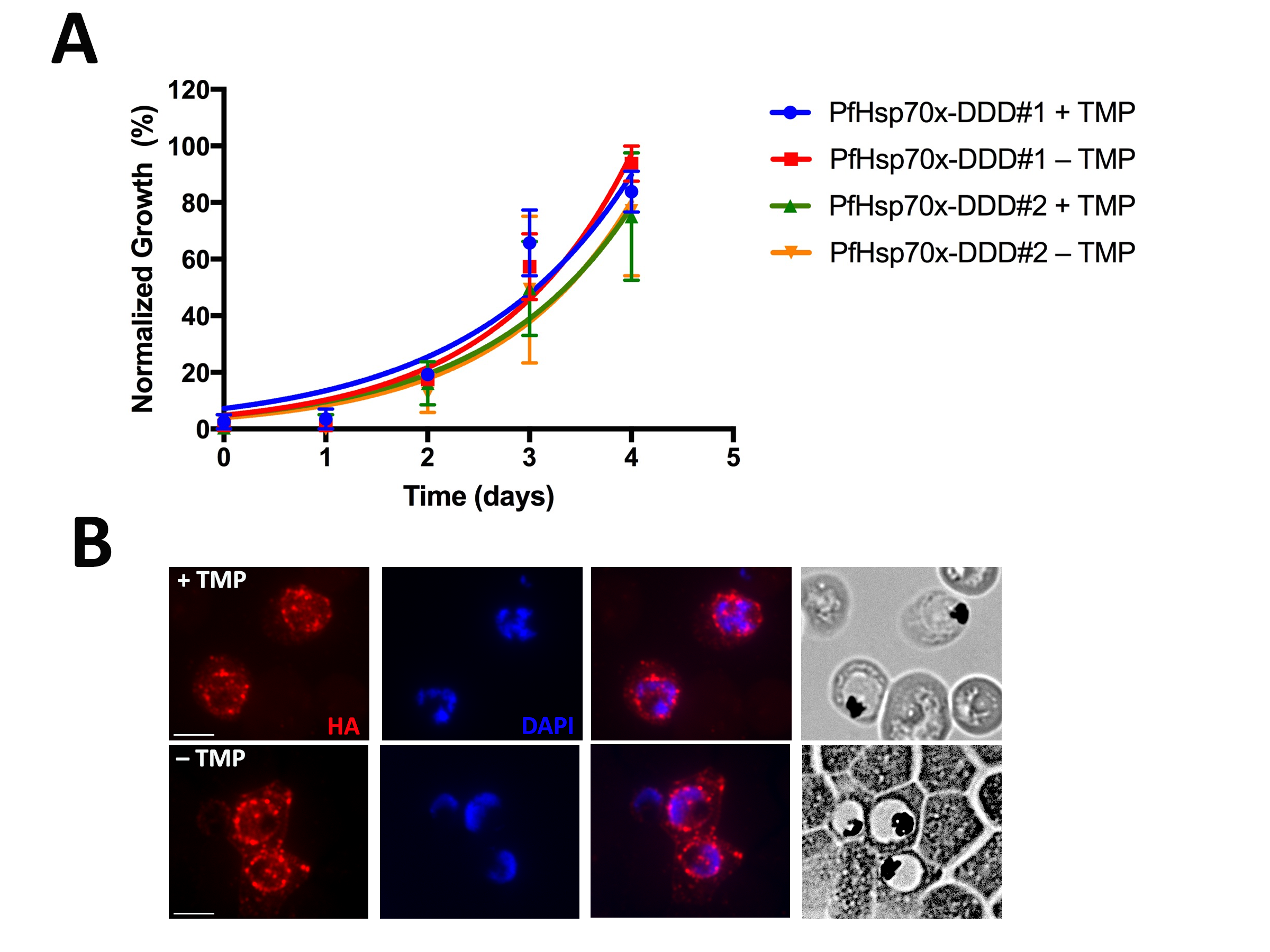

Supplement: FIG S2 [file sph005172367sf2.tif]

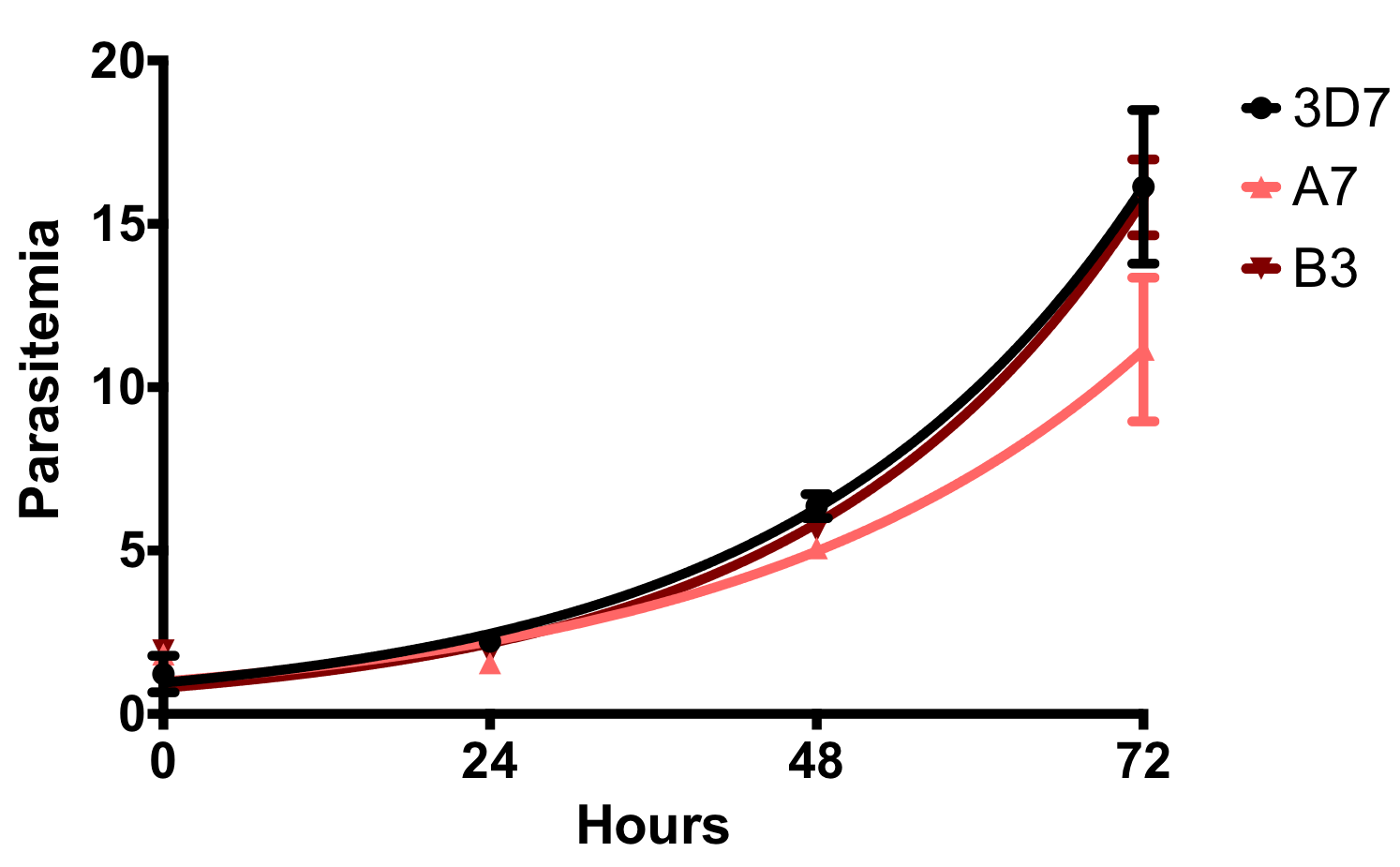

Supplement: FIG S3 [file sph005172367sf3.tif]
